# Supplementary material for: The Systems Biology Research Tool: evolvable open-source software
Source: BMC Syst Biol. 2008 Jun 29;2:55. doi: 10.1186/1752-0509-2-55 (PMC2446383; doi:10.1186/1752-0509-2-55)
Supplement: Additional file 1 — SBRT Archive. An archive of the current version of the Systems Biology Research Tool. [file 1752-0509-2-55-S1.zip › sbrt-1.4.0/doc/developers_guide/api/sbrt/kernel/math/class-use/Polynomial.html]

Uses of Interface sbrt.kernel.math.Polynomial


|  |  |  |
| --- | --- | --- |
| **Overview** \| **Package** \| **Class** \| **Use** \| **Tree** \| **Deprecated** \| **Index** \| **Help** | |  |
| PREV   NEXT | **FRAMES**    **NO FRAMES**     **All Classes** |  |


---


## **Uses of Interface sbrt.kernel.math.Polynomial**

| Packages that use Polynomial | |
| --- | --- |
| **sbrt.kernel.math** | Provides classes and interfaces for creating objects relevant to mathematics. |

Uses of Polynomial in sbrt.kernel.math

| Subinterfaces of Polynomial in sbrt.kernel.math | |
| --- | --- |
| `interface` | `LinearComb<V>`             This interface is used to represent linear combinations. |
| `interface` | `QuadraticExpr<V>`             This interface is used to represent quadratic expressions. |

| Classes in sbrt.kernel.math that implement Polynomial | |
| --- | --- |
| `class` | `AbstractLinearComb<V>`             This abstract class is a skeleton implementation of `LinearComb`. |
| `class` | `AbstractPolynomial<V>`             This abstract class is a skeleton implementation of `Polynomial`. |
| `class` | `HashLinearComb<V>`             This class is a concrete implemenation of `LinearComb`. |

| Methods in sbrt.kernel.math that return Polynomial | |
| --- | --- |
| `Polynomial<V>` | `Polynomial.plugInNumbers(java.util.Map<?,java.lang.Double> variableValues)`             Plugs in the provided numerical values to produce a new polynomial. |

---


|  |  |  |
| --- | --- | --- |
| **Overview** \| **Package** \| **Class** \| **Use** \| **Tree** \| **Deprecated** \| **Index** \| **Help** | |  |
| PREV   NEXT | **FRAMES**    **NO FRAMES**     **All Classes** |  |


---
